# Improved Methods for Reprogramming Human Dermal Fibroblasts Using Fluorescence Activated Cell Sorting

**DOI:** 10.1371/journal.pone.0059867

**Published:** 2013-03-29

**Authors:** David J. Kahler, Faizzan S. Ahmad, Anita Ritz, Haiqing Hua, Dorota N. Moroziewicz, Andrew A. Sproul, Carmen R. Dusenberry, Linshan Shang, Daniel Paull, Matthew Zimmer, Keren A. Weiss, Dieter Egli, Scott A. Noggle

**Affiliations:** 1 The New York Stem Cell Foundation, New York, New York, United States of America; 2 Weill Cornell Medical College in Qatar, Education City, Doha, Qatar; 3 Division of Molecular Genetics, Department of Pediatrics and Naomi Berrie Diabetes Center, Columbia University, New York, New York, United States of America; University of Freiburg, Germany

## Abstract

Current methods to derive induced pluripotent stem cell (iPSC) lines from human dermal fibroblasts by viral infection rely on expensive and lengthy protocols. One major factor contributing to the time required to derive lines is the ability of researchers to identify fully reprogrammed unique candidate clones from a mixed cell population containing transformed or partially reprogrammed cells and fibroblasts at an early time point post infection. Failure to select high quality colonies early in the derivation process results in cell lines that require increased maintenance and unreliable experimental outcomes. Here, we describe an improved method for the derivation of iPSC lines using fluorescence activated cell sorting (FACS) to isolate single cells expressing the cell surface marker signature CD13^NEG^SSEA4^POS^Tra-1-60^POS^ on day 7–10 after infection. This technique prospectively isolates fully reprogrammed iPSCs, and depletes both parental and “contaminating” partially reprogrammed fibroblasts, thereby substantially reducing the time and reagents required to generate iPSC lines without the use of defined small molecule cocktails. FACS derived iPSC lines express common markers of pluripotency, and possess spontaneous differentiation potential *in vitro* and *in vivo*. To demonstrate the suitability of FACS for high-throughput iPSC generation, we derived 228 individual iPSC lines using either integrating (retroviral) or non- integrating (Sendai virus) reprogramming vectors and performed extensive characterization on a subset of those lines. The iPSC lines used in this study were derived from 76 unique samples from a variety of tissue sources, including fresh or frozen fibroblasts generated from biopsies harvested from healthy or disease patients.

## Introduction

The discovery that differentiated somatic cells could be reprogrammed to an embryonic stem cell-like state by forced expression of four transcription factors (Oct4, Klf4, Sox2, cMyc) has revolutionized the stem cell field [1]. Reprogrammed, or induced pluripotent stem cells (iPSCs), show remarkable similarities to embryonic stem cells (ESCs) and hold great promise for *in vitro* disease modeling, drug discovery, and therapeutic interventions because they provide a potentially unlimited source of differentiated cells from individuals with specific diseases [2], [3], [4], [5], [6].

However, initial derivation of stable iPSC clones by viral transduction of dermal fibroblasts is a slow (4–6 weeks) and inefficient (≤0.01% of total fibroblasts) process. Current methods of identifying colonies of *bona fide* iPSCs early in the reprogramming process (2–3 weeks post-infection) utilize light microscopy and manual isolation of candidate colonies, which requires training and expertise in advanced cell culture techniques. To enable future clinical applications requiring *de novo* iPSC derivation, there remains a need for standardized and validated methods for identifying, isolating and purifying reprogrammed cells.

Previous imaging studies based on tracking of cell-of-origin suggest that early events occur during defined factor reprogramming, including a change in cell proliferation rates and morphology [7], downregulation of CD13, a marker of mesenchymal cells including fibroblasts [8], as well as upregulation of the cell surface markers of pluripotency SSEA4 and TRA-1-60 [9]. These studies demonstrate that both partially and fully reprogrammed iPSCs can be identified by combined use of surface expression of multiple markers. Recently, a method of enriching reprogrammed fibroblasts by fluorescence activated cell sorting (FACS) for cells with dual expression of the pluripotency surface markers SSEA4 and TRA-1-81 arising late during reprogramming was described [10]. While a step forward, this method relies heavily on the use of a defined small molecule cocktail, and multiple rounds of sorting and extensive screening to identify fully reprogrammed clones. This suggests that pluripotency markers alone are not sufficient to purify fully reprogrammed iPSCs. Additionally, it is likely that the high variability among clones seen within this population is compounded by the use of integrating vectors to deliver the reprogramming factors. Here, we confirm that throughout the reprogramming process a significant proportion of SSEA4^POS^Tra-1-60^POS^ cells retain the fibroblast surface marker, CD13. Through the use of negative selection against CD13, we were able to purify fully reprogrammed iPSCs from partially reprogrammed cells and parental fibroblasts by FACS. This method removes contaminating cells at an early stage and can be used with a variety of cell populations including: cells reprogrammed with DNA-integrating or non-integrating viruses; fibroblasts harvested from healthy or specific disease patients. Using this method we have generated and characterized 228 iPSC lines from 76 fibroblast lines obtained from multiple sources including fresh biopsies, frozen stocks, and cell line repositories.

## Materials and Methods

### Ethics Statement

The 0819 and 0825 foreskin fibroblast lines were provided from the Skin Disease Research Center Core at Columbia University under notice of institutional review board (IRB) exemption (Columbia University) as no interaction with subjects and no identifiable information were made available to researchers. The 1018 fibroblast line was derived from an upper arm skin biopsy from a 32-year-old female with type I diabetes, diagnosed at age 10. The 1023 fibroblast line was derived from an upper arm skin biopsy from a healthy 23-year-old male. Protocols for obtaining the 1018 and 1023 skin biopsies and their use in reprogramming were approved by the IRB and stem cell research oversight (SCRO) committee of Columbia University and subjects provided signed informed consent [11]. Fibroblast line 10001.131.01(131) was obtained from a skin biopsy harvested from the left temple of a 61-year-old female. Protocols for obtaining the 10001.131.01 skin biopsies and their use in reprogramming were reviewed and approved by the Western Institutional Review Board (WIRB) and subjects provided signed informed consent.

All cell lines summarized in Table S6 were obtained under signed informed consent or are exempt as they were obtained from a Coriell cell repository (http://www.ccr.coriell.org) and no patient contact was made. The approval agency and IRB protocol numbers are as follows: Alzheimer and FTD (Exempt Coriell); Parkinsons; (WIRB NYSCF 10-0001); GAN (AAAE9976 Columbia University); Cardiac LMNA and LongQT (AAAD5685(Y2M00) Columbia University);MODY, T1D, and T2D (AAAD2280 Columbia University); MS_RR(NYSCF 10-005 WIRB ); MS_SP(NYSCF 10-001 WIRB ); healthy control lines were obtained under all protocols listed above.

All mouse studies were reviewed and approved by the institutional animal care and use committee (IACUC) of Columbia University (Protocol Approval Number: AAAD8809). All procedures were performed under anesthesia using 1–5% isofluorane, and all efforts were made to minimize suffering.

### Fibroblast Cell Culture

Explants of 3-mm dermal biopsies were minced and placed in a 60-mm tissue culture dish under a sterile coverslip held down by sterilized silicon grease. Fibroblast medium [Dulbecco's modified Eagle's medium (DMEM), supplemented with 10% fetal bovine serum, Glutamax™, and penicillin/streptomycin (Invitrogen, Carlsbad, CA)] was added, and dishes incubated at 37°C in a humidified 5% CO_2_ atmosphere with media exchange every 5 days. Fibroblast outgrowths were harvested by trypsinization, expanded in a T25 flask in fibroblast medium, and allowed to reach ∼90% confluence prior to freezing or splitting for reprogramming as described below. For reprogramming, fibroblasts were used within the first three passages from biopsy or within one passage after a thawing. All parent fibroblast and reprogrammed lines were subjected to cytogenetic analysis (Cell Line Genetics) and for DNA fingerprinting by short tandem repeat (STR) analysis.

### Fibroblast Reprogramming

Fibroblasts were reprogrammed using high titer stocks of vesicular stomatitis virus G (VSVG)-coated retroviruses containing Oct4, Sox2, cMyc, and Klf4 (Harvard Gene Therapy Initiative at Harvard Medical School) as previously described [12], or the non-integrating CytoTune™ - Sendai viral vector kit (Life Technologies, A13780). Fibroblasts reprogrammed with retroviruses were infected at 1×10^4^ cells/well in 1 ml of human ESC medium (HUESM) [Knockout™ DMEM supplemented with 20% Knockout™ serum replacement (Invitrogen), 10 ng/ml bFGF (Invitrogen), nonessential amino acids (Invitrogen), β-mercaptoethanol (Invitrogen), L-glutamine, and penicillin/streptomycin (Invitrogen)]. The medium was exchanged on day 2 to HUESM containing ALK5 inhibitor SB431542 (2 µM; Stemgent), MEK inhibitor PD0325901 (0.5 µM; Stemgent), and ROCK inhibitor [13] Thiazovivin (0.5 µM; Stemgent)] and changed daily thereafter. For Sendai virus mediated reprogramming, 5×10^5^ fibroblasts were infected in fibroblast medium at a multiplicity of infection of 3 (MOI3) for two days with daily (HUESM) media exchanges. At day 7–10 days post-infection by retro or Sendai viral protocols, cells were subjected to FACS analysis or passaged onto feeder cells by enzymatic dissociation using Dispase (GIBCO) and/or Accutase (Sigma-Aldrich) then passaged onto γ-irradiated murine embryonic fibroblasts (MEFs; Globalstem) or Matrigel™ (BD Biosciences) coated plates in HUESM at 2×10^3^ cells/cm^2^.

### Fluorescent Activated Cell Sorting of Reprogrammed Fibroblasts

Cells enzymatically harvested as described above were filtered through a 35 µm cell strainer (BD biosciences) to obtain a single cell suspension prior to resuspension in 100 µl of a sterile iPSC staining buffer [DPBS containing 0.5% bovine serum albumin fraction V (BSA; Invitrogen), 100 U/ml penicillin/streptomycin (Invitrogen), 2 mM EDTA (Invitrogen), and 20 mM glucose (Sigma)]. A cocktail of fluorescence-conjugated antibodies [1 µl each anti-CD13 (555394) anti-SSEA4 (560219) and anti-Tra-1-60 (560173), obtained from BD, CA] was added to the cells and incubated at room temperature (RT) for 15 minutes shielded from light. Stained cells were washed once with iPSC staining buffer and sorted immediately on a 5 laser BD Biosciences ARIA-IIu™ SOU Cell Sorter using “gentle FACS” sorting conditions based on the work of Pruszak *et al*. (100 µm ceramic nozzle, 20 psi) [14]. Some experiments included antibodies against SSEA3 (BD, 560237), or CD326 (BD, 347200) in the cocktail to confirm the pluripotent status of the reprogrammed cells. Target cell populations were sorted directly onto MEF layers (ARIA plate holder at 37°C) at between 2×10^3^ and 5×10^4^ cells/well in a 6-well plate containing HUESM plus 20 µM Y-27632 (ROCK inhibitor; Calbiochem). Two days after sorting, the ROCK inhibitor was removed from the medium and replaced with SB431542 (2 µM), PD0325901 (0.5 µM), and Thiazovivin (0.5 µM) [13] with daily media exchange. Several individual colonies were picked 7–10 days after sorting and expanded for characterization.

### Quantitative RT-PCR

Total RNA was isolated from duplicate or triplicate cell samples using the RNeasy kit (QIAGEN, 74136). cDNA synthesis was performed on 1 µg RNA with SuperScript™ III First-Strand system (Invitrogen 18080-051) and oligo(dT) primers. The cDNA was diluted to a final volume of 200 µl and 1 µl was added to 500 nM of the forward and reverse primers in a final volume of 10 µl per PCR reaction. Quantitative real-time PCR was performed using the GoTaq® SYBR Green Master kit (Promega, A6001) and Mx3000p QPCR system (Stratagene). Primer sequences are provided in **Table S1.**

### Southern Blotting

Probes for human Oct4, Sox2, and KLF4 were generated by PCR using the digoxigenin (DIG) probe synthesis kit (Roche) and Southern blotting was performed using DIG System detection reagents (Roche). Genomic DNA was isolated from human ESCs, parent fibroblast cells, and iPSCs using the Qiagen DNA Mini kit. DNA samples (5–10 µg) were digested overnight with BglII to generate a single cut in the integrated viral backbone of each transgene, and digests were resolved on a 0.8% agarose gel (without ethidium bromide), which was then denatured with 0.5% NaOH and neutralized. The gel was transferred to a nylon membrane by overnight capillary transfer. Wet membranes were crosslinked with 120 mJ UV (HL-2000 Hybrilinker, UVP) and allowed to dry. Membranes were pre-hybridized with DIG Easy Hyb buffer for at least 1 h at 55°C, then incubated with the appropriate probe overnight at 55°C. Membranes were washed thoroughly using the DIG Wash and Block kit, blocked for at least 1 h, and incubated with anti-DIG antibody for 30 min. Membranes were then washed and treated with CDP-Star reagent to detect DIG-incorporated bands. Blots were stripped and re-probed according to the manufacturer’s instructions. Probe sequences are provided in **Table S2.**

### NanoString nCounter Assay

Total RNA was isolated from each iPSC clone between passage 10 and 15 using the RNeasy kit (Qiagen). A 100-ng sample of RNA was then profiled using the NanoString nCounter system (NanoString, Seattle, WA) using one of two custom-designed codesets. The pluripotency codeset contains 25 probes for detection of the Sendai and retroviral transgenes, pluripotency and spontaneous differentiation markers, and housekeeping genes **Table S3.** The lineage codeset is derived from a previous study [15] and contains 85 probes for the three germ layers in addition to probes for retroviral and Sendai transgenes, and housekeeping genes **Table S4.** RNA from a retrovirus-positive or Sendai-positive control line, a fibroblast line (1043), and two human ESC lines (HUES42 and Hues16) was included in each run. Data were analyzed using the nSolver Analysis Software v1.0 (NanoString) and plotted using Prism (Graphpad Software, La Jolla, CA). Data quality control and normalization to geometric mean for both internal positive controls, and subsequently for housekeeping genes, was performed in the nSolver analysis software.

### Embryoid Body Formation

Embryoid bodies (EB) were formed by placing clumps of iPSCs in 96-well non-tissue culture treated V-bottom plates (Evergreen 222-8031-01V) containing HUESM. After 5 weeks of culture, EBs were harvested, fixed in 4% paraformaldehyde (PFA) for 30 min at RT and processed in 15% and 30% sucrose solutions for one day each prior embedding in O.C.T., freezing, sectioning into 10 µm slices and mounting on glass slides. EB sections were immunostained with antibodies against the markers shown in **Table S5.** Briefly, EB sections were incubated with blocking solution 10% donkey serum in PBST (PBS with 0.1% Triton-100) for 1 h at RT, followed by an overnight incubation at 4°C with primary antibodies. After washing three times with PBST, sections were incubated for 1 h at RT with appropriate secondary antibodies obtained from Molecular Probes. Finally, sections were washed and counterstained with DAPI (1∶1000 in PBS) for 15 min at RT.

### Teratoma Assay

Manually (1023_C) and FACS-derived (1023_D2) cells were dissociated using Dispase (Gibco 17105-041) for 15 minutes at 37°C to produce small clumps containing approximately 100–200 iPSCs/clump. The clumps were suspended in 100 µl of HUESM containing 100 µl Matrigel™ (BD Biosciences) and injected subcutaneously into NOD-SCID *Il2rg*-null mice (Jackson Laboratory) following an intraperitoneal injection of carprofen (Pfizer) at 5 mg/kg. Teratomas were allowed to grow for 6–8 weeks, isolated by dissection and fixed in 4% PFA overnight at 4°C. Fixed tissues were embedded in paraffin, sectioned at 10 µm and stained with hematoxylin and eosin (H&E).

## Results

### FACS Derivation of iPSCs

Previous studies have demonstrated downregulation of the human fibroblast marker CD13 [8], and upregulation of the pluripotency markers SSEA4 and TRA-1-60 occurs during reprogramming [9]. These studies suggest that isolation of fully reprogrammed iPSCs during early stages of reprogramming may be accomplished by FACS using a combination of positive and negative surface markers. While current sorting strategies for purification of pluripotent cells rely on positive selection [7], it is possible that a significant proportion of clones isolated using this method may not be fully reprogrammed. To test this hypothesis we first optimized the conditions for survival of live-cell sorted, fully reprogrammed cells by examining the expression levels of three surface markers in a manually derived, early passage clone (p4) of an iPSC line (1018) cultured on MEFs.

**Figure 1:**
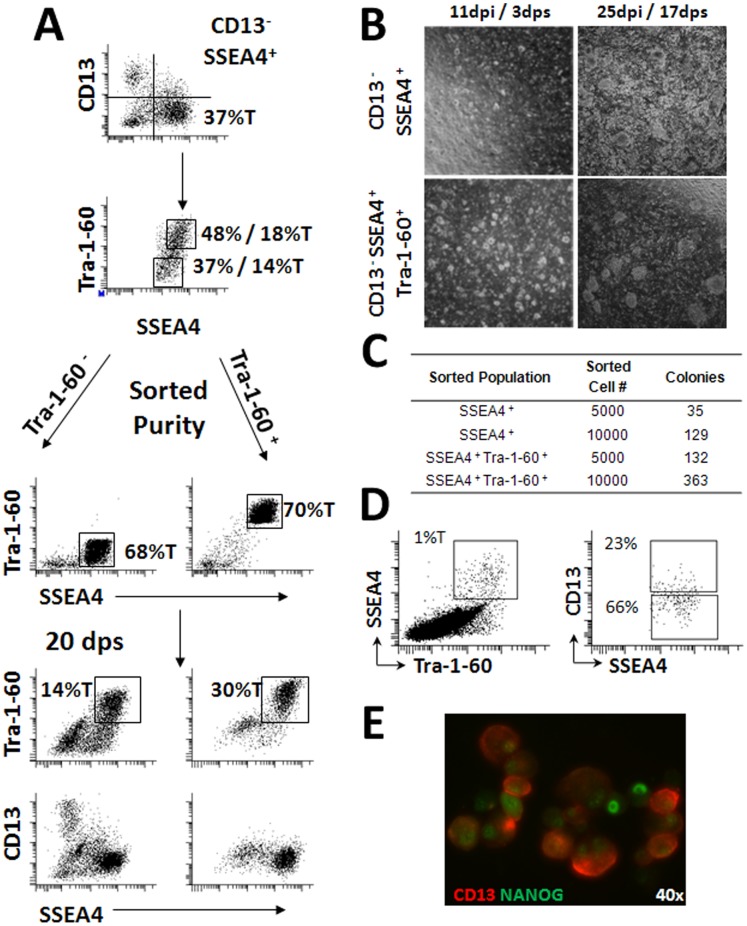
Enhanced derivation and maintenance of virally reprogrammed fibroblasts using Fluorescence Activated Cell Sorting. (**A**) CD13^NEG^SSEA4^POS^Tra-1-60^NEG^ and CD13^NEG^SSEA4^POS^Tra-1-60^POS^ populations from the manually derived 1018 clone were sorted onto MEF feeder layers and expanded for 20 days prior to reanalysis by flow cytometry to assess retention of sorted surface markers. dpi = days post infection. dps = days post sort. (**B**) CD13^NEG^SSEA4^POS^ and CD13^NEG^SSEA4^POS^Tra-1-60^POS^ populations were sorted onto MEF layers at seven days post infection and imaged at 3 and 17 dps to assess colony formation. (**C**) Colony counts arising from the sorted cell populations shown in Panel B at 17 dps (25 dpi). (**D**) Gating structure used in the analysis of CD13^POS^ cells present within the SSEA4^POS^Tra-1-60^POS^ population at 7 dpi. (**E**) Fluorescence microscopy demonstrating NANOG expression in CD13^POS^ cell at 7 dpi. 40× magnification. CD13 shown in red. Nanog in shown Green. Values designated %T indicates proportion of total cells within the culture positive for the indicated combinations of surface markers. Values without T designation indicate the proportion of CD13^NEG^SSEA4^POS^ cells that are Tra-1-60^POS^ or Tra-1-60^NEG^ in Panel A and D.

Populations of cells expressing all three markers were found in the culture suggesting a heterogeneous mixture of cells containing parental and partially reprogrammed fibroblasts **Figure 1A**. We then sorted the CD13^NEG^SSEA4^POS^Tra-1-60^POS^ (Tra-1-60^POS^) population and, as a control, the CD13^NEG^SSEA4^POS^Tra-1-60^NEG^ (Tra-1-60^NEG^) population to approximately 70% purity directly into one well of a 6 well plate containing MEFs in the presence of ROCK inhibitor Y-27632. The sorted populations were maintained for 20 days on MEFs without ROCK inhibitor or removal of differentiated cells or splitting prior to reanalysis by flow cytometry (FCM). At 20 days post-sorting (dps), the cultures originating from the enriched Tra-1-60^POS^ population contained fewer Tra-1-60^NEG^ differentiated cells and no detectable CD13^POS^ parental fibroblasts. The Tra-1-60^POS^ population was present in these cultures at approximately double the proportion found in the Tra-1-60^NEG^ enriched culture (30% vs. 14%). Conversely, at 20 dps the culture originating from the Tra-1-60^NEG^ enriched population contained a Tra-1-60^POS^ population at a similar proportion to the originally sorted culture (18%T vs. 14%T). However, these cultures also contained a higher proportion of differentiated or transformed cells (CD13^NEG^SSEA4^NEG^Tra-1-60^NEG^) as well as adult fibroblasts (CD13^POS^) suggesting that our FACS conditions allow for purification of fully reprogrammed cells from contaminating cell types.

To confirm this strategy, adult skin fibroblasts at 8 days post infection (dpi) were sorted based on the CD13^NEG^SSEA4^POS^Tra-1-60^POS^ surface marker profile. As a control, the CD13^NEG^SSEA4^POS^ population that contained two subpopulations of cells expressing either Tra-1-60^POS^ or Tra-1-60^NEG^ was isolated in parallel. 5,000 or 10,000 cells from each population were sorted directly into MEF-containing plates, and monitored for the formation of colonies. Small but distinct colonies were evident in both sorted populations as early as 3 days post sort (dps), with the CD13^NEG^SSEA4^POS^Tra-1-60^POS^ population producing larger and more abundant colonies than the CD13^NEG^SSEA4^POS^ population (3 dps, 11 dpi; **Figure 1B**). Following an additional 2 weeks of expansion without manual removal of differentiated cells, wells containing the CD13^NEG^SSEA4^POS^ population had become overgrown with transformed and differentiated cells, whereas wells containing the sorted CD13^NEG^SSEA4^POS^Tra-1-60^POS^ cells contained large, well-separated colonies with few differentiated cells between the colonies and lacked cells with transformed morphology (17 dps, 25 dpi**;** **Figure 1B**). We observed a 3–4 fold increase in the number of colonies present in wells containing the sorted CD13^NEG^SSEA4^POS^Tra-1-60^POS^ cells compared to the sorted CD13^NEG^SSEA4^POS^ cells **Figure 1C**.

We then analyzed CD13 expression within the SSEA4^POS^Tra-1-60^POS^ population of reprogrammed fibroblasts at 7 dpi. Roughly one quarter of the SSEA4^POS^Tra-1-60^POS^ population expressed CD13 indicating the presence of a heterogeneous population of fully reprogrammed, transformed, or transitioning cells (23% CD13^POS^, 66% CD13−; **Figure 1D**), some of which expressed both Nanog and CD13 **Figure 1E**.

**Figure 2:**
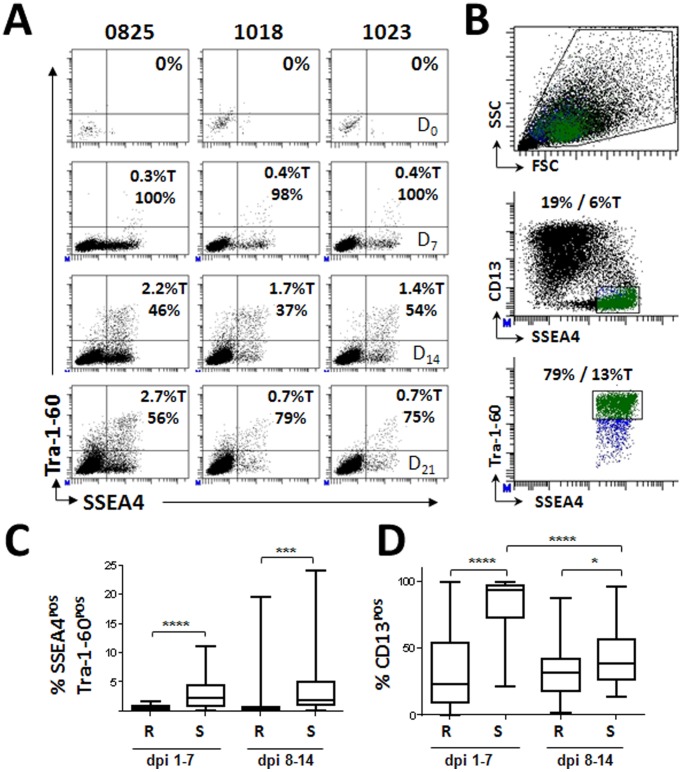
Fibroblasts undergoing viral reprogramming exhibit characteristic expression levels of surface markers at early time points post infection. (**A**) Foreskin (0825) and adult dermal fibroblast (1018 and 1023) lines underwent four factor retroviral reprogramming and were analyzed by flow cytometry for the emergence of the CD13^NEG^SSEA4^POS^Tra-1-60^POS^ population at seven day intervals post infection. Values designated %T indicates proportion of total cells within the culture positive for the indicated combinations of surface markers. Values without T designation indicate the proportion of cells positive within the parent gate. (**B**) Gating structure used to sort the CD13^NEG^SSEA4^POS^Tra-1-60^POS^ populations for all cell lines derived in this study. Live cell are first defined using forward (FSC) and Side (SSC) light scattering properties. The CD13^NEG^SSEA4^POS^ population is then selected from the live cell gate (blue cells). The highest Tra-1-60^POS^ expressing cells are then selected from the CD13^NEG^SSEA4^POS^ population (Green cells) and sorted for expansion and characterization. (**C**) Comparison of SSEA4^POS^Tra-1-60^POS^ populations present in Retro (R) or Sendai (S) viral infected fibroblast cultures during first two weeks of programming. (**D**) Comparison of CD13^POS^ cells present within the SSEA4^POS^Tra-1-60^POS^ populations during first two weeks of programming following Retro (R) or Sendai (S) infection. Dpi 1–7:(R) n = 29, (S) n = 21. Dpi 8–14: (R) n = 32, (S) n = 46. Total n = 228. Statistical significance was assessed via Student’s t-Test. * p< = 0.05, ** p< = 0.001, *** p< = 0.001, ****p< = 0.0001.

Because surface marker expression during reprogramming is dynamic, we wanted to first identify the earliest time point at which to enrich fully reprogrammed iPSCs. Time course analysis conducted by flow cytometry following retroviral reprograming suggested that SSEA4^POS^Tra-1-60^POS^ cells were detectable as early as 7 dpi, and their proportion increased up to 21 dpi, then remained constant as marker negative cells outgrew the reprogrammed cells **Figure S1**. The SSEA4^POS^Tra-1-60^POS^ population also expressed the SSEA3 and CD326 pluripotency markers [16], [17], [18], [19]. SSEA4^POS^CD13^POS^ cells appeared by 7 dpi and while most of this population disappeared by 14 dpi, a proportion remained in the culture. To test whether this timing was consistent among different skin samples, we analyzed cultures derived from a foreskin fibroblast line (0825), a healthy adult control fibroblast line (1023), and a fibroblast line from a subject with type I diabetes (1018) for up to 21 dpi. The three fibroblast cell lines showed a consistent emergence of pluripotent surface markers with SSEA4^POS^Tra-1-60^POS^ cells present at low numbers at 7 dpi (D_7_, 0.3%–0.4% **Figure 2A**), increasing in proportion at 14 dpi (D_14_, 1.4%–2.2%), and decreasing by day 21 as other cells overtook the culture (D_21_, 0.7%), suggesting a consistent appearance of potentially early reprogrammed cells between 7 and 14 dpi. However, at early time points, the majority of SSEA4^POS^Tra-1-60^POS^ cells also expressed CD13 (D_7_, 98%–100%). The proportion of CD13^POS^SSEA4^POS^Tra-1-60^POS^ decreased approximately half by day 14 post infection (D_14_, 37%–54%), suggesting loss of this fibroblast marker on cells undergoing reprogramming. Interestingly, the CD13^POS^SSEA4^POS^Tra-1-60^POS^ population increased again by day 21, suggesting that this population was expanding or that CD13^NEG^ cells were lost from the culture.

Based on these results we developed the sorting strategy shown in **Figure 2B** which omits the contaminating partially reprogrammed CD13^POS^SSEA4^POS^ population by selecting the highest Tra-1-60^POS^ expressing cells within the CD13^NEG^SSEA4^POS^ population. Using this strategy we FACS derived 228 individual iPSC lines from over 75 fresh or frozen fibroblast lines generated from biopsies harvested from healthy or disease patients using either integrating (retroviral) or non-integrating (Sendai virus) reprogramming vectors and performed extensive characterization on a subset of those lines which is described in the data that follows. We further characterized the first 2 weeks of the reprogramming process on 128 FACS derived iPSC lines using the analysis structure shown in **Figure 1D**. As shown in **Figure 2C**, a higher percentage of SSEA4^POS^Tra-1-60^POS^ cells were generated in Sendai infections compared to retroviral infections over the entire time course. However, Sendai infections demonstrated a delayed reduction in the proportion of CD13^POS^SSEA4^POS^Tra-1-60^POS^ cells **Figure 2D**. By the second week of induction, the proportion of the CD13^POS^ population between the cultures was similar.

### FACS Derived Lines are Pluripotent

To further characterize this defined selection strategy, we compared the phenotype and function of the FACS-derived iPSC clones to manually picked clones. First, the 0825, 1018, and 1023 fibroblast lines shown in **Figure 2A** reprogrammed using the 4-factor retroviral protocol were subjected to either FACS derivation at 7 dpi or standard picking techniques. For each method, one clone from each line was randomly selected for expansion and further characterization by a standard battery of assays, including karyotypic analysis, DNA fingerprint, pluripotent surface marker expression, qRT-PCR, and Embryoid body (EB) and teratoma formation. All fibroblasts and reprogrammed iPSC lines displayed normal karyotypes, and had DNA fingerprints matching the parental fibroblast line **Figure S2**. Clones from the 0825 foreskin fibroblast line had a DNA fingerprint that matched a major subpopulation in the parental fibroblasts that contained a contaminating subpopulation with a different genotype, suggesting isolation of clonal cultures from a mixed population.

**Figure 3:**
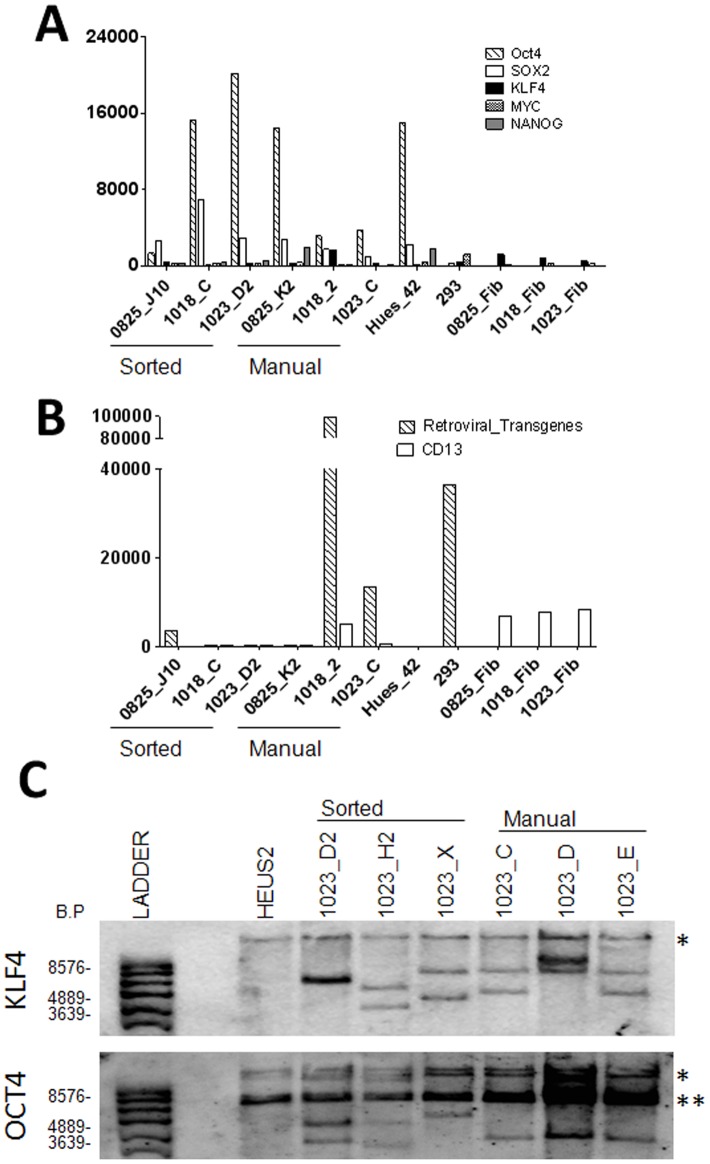
Fluorescence Activated Cell Sorting generates higher quality independent clones than manual derivation. Modified pluripotent scorecard assay was performed on manually and FACS derived clones to demonstrate (**A**) activation of endogenous gene expression and (**B**) silencing of gene expression and presence of unreprogrammed and transformed fibroblasts CD13^POS^ in manually derived clones. (**C**) Three sorted and three picked lines from patient 1023 were used to compare the ability of both methods to generate independent clones. 10 μ of genomic DNA were cut overnight with BglII and submitted to Southern blotting. The HUES line HES2 was used as a positive control for endogenous KLF4/OCT4, and as a negative control for transgene insertions. Samples were first blotted for KLF4, then stripped and reblotted for OCT4. Picked clones 1023 C and E are consistent with being the same clone by both KLF4 and OCT4 blotting. * indicated the predicted endogenous KLF4/OCT4 bands, and ** indicated a consistent band found in all samples blotted with OCT4.

Both the manually and FACS-derived iPSC lines expressed common markers of pluripotency, including the surface marker Tra-1-60 and the transcription factor Nanog, and generated compact colonies morphologically consistent with normal hESCs, **Figure S3 (A–B)**. Next, the cell lines were expanded for ten passages and the expression of endogenous Nanog, Oct4, Sox2, cMyc, and Klf4, and silencing of the viral transgenes Oct4, Sox2, Klf4, and cMyc were assayed. We used a probe-based Nanostring nCounter transcript quantification assay, to assess pluripotency by detecting both activation of endogenous gene expression **Figure 3A** and silencing of retroviral transgenes **Figure 3B**. These data were further confirmed by qPCR **Figure S3** and revealed a similar pattern of endogenous gene expression in all iPSC lines compared to undifferentiated hESC controls **Figure 3A** **, Figure S3.** However, two of the three manually derived clones (1018_2 and 1023_C) maintained much higher expression of the viral transgenes than the sorted clones **Figure 3B**. Additionally, the 1018_2 cultures expressed CD13, indicating the presence of non-reprogrammed or partially transformed human fibroblasts in the manually picked lines **Figure 3B**. These analyses suggest that selection of single cells based on CD13^NEG^SSEA4^POS^Tra-1-60^POS^ expression can be used to select against partially reprogrammed or contaminating cell types in reprogrammed cultures. The full data set for these experiments is provided in **Table S7.**

**Figure 4:**
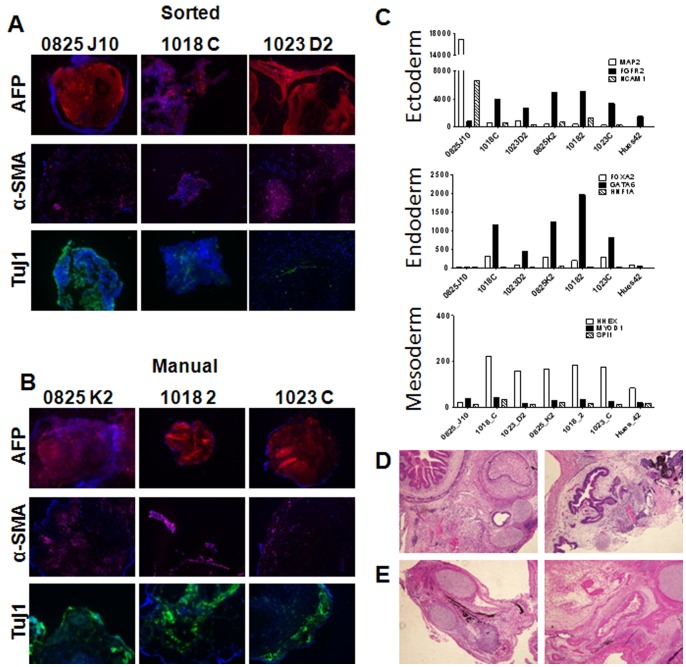
hIPSC lines derived by Fluorescence Activated Cell Sorting possess *in vitro* and *in vivo* spontaneous differential potential. Embryoid bodies were derived from FACS (**A**) or manually derived clones (**B**) and stained for expression of alpha fetoprotein, smooth muscle actin and beta III tubulin (Tuj1) to demonstrate differentiation potential *in vitro* potential. 10× Magnification (**C**) Differentiation potential of the derived lines for expression of germ layer genes present in the Lineage scorecard assay. (**D**) Teratomas from FACS (**D**) or manually derived (**E**) clones of 1023 fibroblast line indicating *in vitro* differentiation potential by formation of three germ layers.

We next examined undirected EB formation to measure the *in vitro* differentiation potential of FACS and manually-derived iPSCs clones. Following differentiation for 5 weeks, EBs were collected and assayed for markers of three embryonic germ layers endoderm, mesoderm, and ectoderm by immunohistochemistry for α1-fetoprotein (AFP), smooth muscle actin (αSMA), and beta III tubulin (Tuj1), respectively. EBs derived from FACS or manually picked clones expressed markers associated with formation of the three germ layers **Figure 4A–B** **.** To further define the differentiation potential of the derived lines, RNA from the EBs were collected after two weeks of differentiation and tested against a panel of lineage-specific nCounter probes **Table S4** previously validated to detect expression of genes commonly found in the three germ layers [15] **Figure 4C**. With the exception of the FACS-derived 0825 line, all lines expressed comparable levels of the germ layer-associated genes, indicating they have similar potential to spontaneously differentiate *in vitro* into any germ layer. The full data set for these experiments is provided in **Table S8.**

To measure the *in vivo* differentiation potential of the iPSCs, immunocompromized mice were injected with FACS or manually derived clones from the 1023 fibroblast line. The resulting teratomas were sectioned and examined by H&E staining. This analysis showed that teratomas generated from sorted **Figure 4D** or manually derived **Figure 4E** clones formed all three germ layer tissues, including gut-like epithelial tissues (endoderm), cartilage (mesoderm), and retinal pigment epithelium (ectoderm). Together, these analyses validate the use of CD13^NEG^SSEA4^POS^Tra-1-60^POS^ expression as a surface marker signature compatible with FACS that can be used to isolate a population of fully reprogrammed iPSCs.

### FACS Derivation Produces Independent Clones

Because iPSC lines can arise from rare apparently stochastic events at early time points during reprogramming, it was important to establish that FACS sorting could isolate multiple independent reprogramming events. To determine whether FACS derivation can produce unique cell lines in a similar manner to manual picking, we performed Southern blotting on several clones derived by both methods. Klf4 and Oct4 probes were used to identify the endogenous and virally integrated forms of the genes. Different banding patterns indicate differences in the chromosomal integration sites and, in some cases, varying numbers of detectable integration events. As shown in **Figure 3C**, all three sorted clones from line 1023 have different integration sites for both Klf4 and Oct4, demonstrating they are independent clones. In contrast, two of the three picked clones from line 1023 have identical banding patterns for Klf4 and Oct4, suggesting they are the same clone. Of the iPSC lines generated from three different fibroblast lines, 8/9 FACS-derived lines were independent clones, while 7/9 manually picked lines were independent (data not shown), suggesting equivalent ability to generate clonal cultures. Therefore, FACS sorting between 7–14 dpi using of CD13^NEG^SSEA4^POS^Tra-1-60^POS^ can generate independent clonal cultures following retroviral reprogramming.

### FACS Derived Lines are Stable at Later Passages

**Figure 5:**
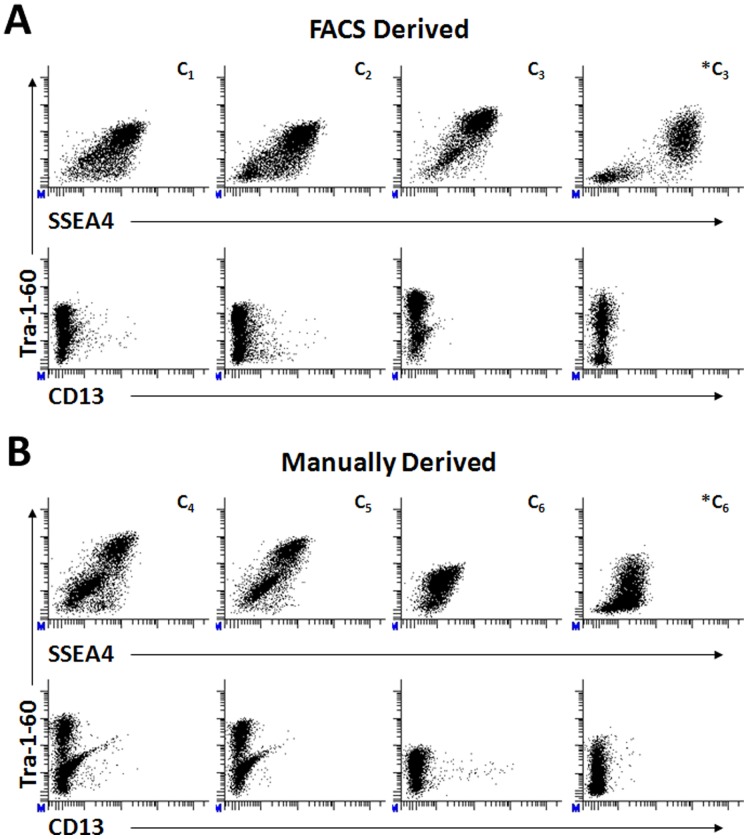
Stability of Fluorescence Activated Cell Sorted and Manually Derived IPSC Lines. Three individual clones were selected from foreskin (0819) fibroblasts lines which previously underwent four factor retroviral reprogramming and were derived by either FACS (**A, C_1_–C_3_**) or manual (**B, C_4_–C_6_**) techniques were analyzed by flow cytometry for pluripotent surface marker expression following expansion on murine embryonic fibroblasts for 12–14 passages. Clones C3 and C6 were adapted to Matrigel and mTSER media around passage11 and expanded for several passages prior to surface marker analysis by flow cytometry to demonstrate stability following changes in culture conditions. Events displayed in the 2D scatter plots are “live” cells as defined by forward and side scatter properties expressing indicated surface markers.

To demonstrate the stability of FACS derived ips clones at later passages, we retrovirally reprogrammed a foreskin fibroblast line (0819) using both FACS and manual derivation methods. Three individual clones were chosen from each derivation method and expanded on MEFs to asses pluripotent surface marker expression by FCM at later passages. As shown in the first row of **Figure 5A–B**, cultures of all clones (C_1_–C_6_) resulting from each derivation method possessed populations of cells positively expressing both SSEA4 and Tra-1-60 at varying proportions indicating stability at between 12–14 passages. Although not shown in **Figure 5**, cultures of these clones were stable at earlier (p4–p11) and later (p20–p25) passages. Clones C_3_ and C_6_ were adapted to matrigel and mTser media (*C_3_ and *C_6_ in Fig. 5A,B) following 11 passages on MEFS and expanded for 3–5 passages to demonstrate the stability of FACS derived ips lines following changes in substrate and media conditions. FCM analysis of Matrigel adapted ips lines show stable surface marker expression with less SSEA4^POS^Tra-1-60^NEG^ populations than the manually derived clone C6. Small populations of CD13^POS^ expressing both Tra-1-60^POS^ and Tra-1-60^NEG^ (second row of **Figure 5A–B**) were present in all cultures with the exception of the FACS derived C_3_ and *C_3_ clones indicating the variability present in individual clones derived under DNA integrating reprogramming techniques. Similar results are observed within clones derived using the non-integrating Sendai viral platform (Data not shown). These results demonstrate that FACS derived ips clones remain stable over multiple passages and following adaptation to feeder free conditions.

### Utility for Multiple Reprogramming Methods

To further validate that the FACS surface marker panel can be used for multiple reprogramming methods, we reprogrammed fibroblasts using non-integrating Sendai viral constructs carrying the four Yamanaka reprogramming factors and compared the FACS and manual derivation methods to determine if there were differences between the integrating and non-integrating reprogramming systems. For these studies, an adult fibroblast line (131) was infected and subjected to either FACS sorting at 11 dpi or to manual derivation. At 11 dpi, the fraction of SSEA4^POS^TRA-1-60^POS^ cells that were also CD13^POS^ was significantly lower (1–2%, data not shown) than with retroviral reprogramming (37–54%, **Figure 2C**), suggesting an accelerated rate of reprogramming. As before with the retroviral lines, several clones from each derivation technique were selected and expanded for characterization following confirmation that the parent fibroblast line possessed a normal karyotype and DNA fingerprint, and was free of contaminating cell lines **Figure S2**.

**Figure 6:**
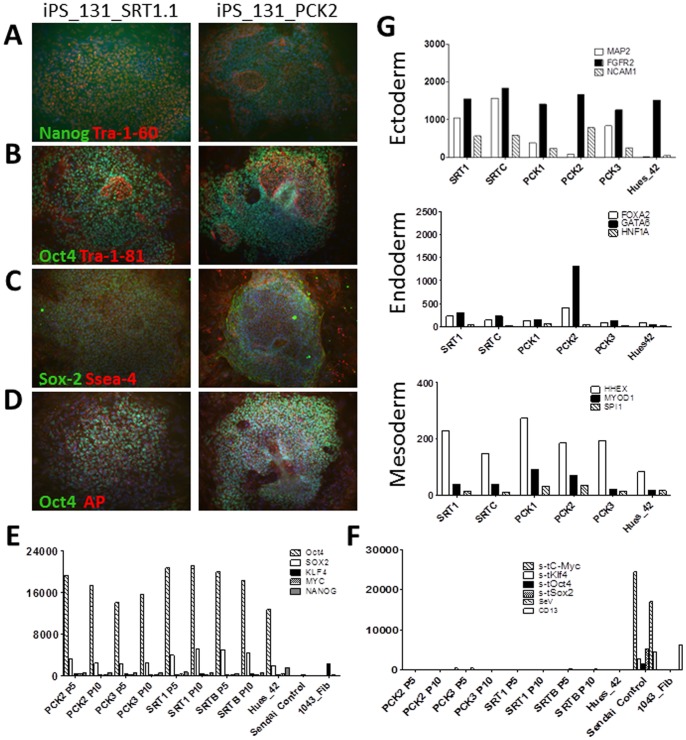
Characterization of Fluorescence Activated Cell Sorted and Manually Derived IPSC Lines by Sendai virus. Immunofluroescence microscopy of the 1001.131.01 line demonstrating expression of common markers of pluripotency by FACS or Manually Derived IPSC lines. Nuclear Transcription Factors shown in Green, Surface Markers shown in Red, Nucleus stained with DAPI in Blue (**A**) Nanog/Tra-1-60 (**B**) Oct4/Tra-1-81 (**C**) Sox2/SSEA4 (**D**) Oct4/Alkaline Phosphatase. 10× Magnification (**E**) Expression of endogenous pluripotent transcription factors (**F**) Silencing of viral transcription factors occur by passage 5. (**G**) Expression levels of transcription factors common to the indicated germ layers from EB generated by the indicated IPSC lines.

As before, individual clones selected from both FACS and manual derivation techniques expressed the common markers of pluripotency, as revealed by immunostaining **Figure 6A–D**. In addition, the sorted (SRT) and picked (PCK) clones showed comparable levels of endogenous Oct4, Sox2, KLF4, MYC, and Nanog gene expression as early as passage 5, which remained relatively constant to passage 10, **Figure 6E**. Similarly, though greatly reduced compared to control infected fibroblasts, Sendai virus gene expression was slightly above background at passage 5 in one sorted and one picked clone. However, this Sendai gene expression was eliminated by passage 10 in both cases, **Figure 6F**. The full data set for these experiments is provided in **Table S9.**

The *in vitro* differentiation potential of FACS and manually derived iPSC clones reprogrammed by the Sendai virus protocol was evaluated by EB formation and a lineage specific nCounter assay as above. Similar trends in gene expression were observed for clones derived under both methods, **Figure 6G**. Both sorted and picked lines expressed levels of the ectodermal marker FGFR2 comparable to that of the control HUES42 line but higher levels of the mesodermal marker HHEX. Most clones showed levels of endodermal gene expression comparable to the HUES42 line with the exception of a manually derived clone that expressed higher levels of GATA6 than the remaining picked lines or FACS-derived lines. The full data set for these experiments is provided in **Table S10.** Collectively, these data demonstrate that using FACS to purify the CD13^NEG^SSEA4^POS^Tra-1-60^POS^ cells from either retroviral or Sendai viral 4-factor reprogramming protocols consistently produces high quality iPSC lines.

## Discussion

Clinical application of iPSC technology will require standardized and reproducible methods for each step of derivation and differentiation into relevant cell types. We have found that the majority of manually derived iPSC lines contain CD13^POS^ cells even after prolonged culture suggesting that these lines were either not fully reprogrammed or that CD13^POS^ cells were carried over during passage. While both manual picking and FACS sorting methods [10] have been used to isolate reprogrammed pluripotent cells, we demonstrated that inclusion of a negative selection marker such as CD13 has significant advantages in improving the purity of reprogrammed cultures. This fact was previously demonstrated [13]. Here, we have validated a surface marker profile that enables selection of early reprogrammed iPSCs following reprogramming with either DNA-integrating or non-integrating viruses by FACS. Employing this strategy as early as 7 dpi isolates a highly purified starting population of fully functional CD13^NEG^SSEA4^POS^Tra-1-60^POS^ cells that are depleted of contaminating non-transduced and transformed fibroblasts. We have successfully generated and characterized 228 individual iPSC lines in 2 years from 76 fibroblast lines obtained from fresh biopsies, frozen stocks, and cell line repositories harvested from healthy and individuals possessing various forms of diabetes, neurodegenerative, cardiac and autoimmune diseases. **Table S6.**

Moreover, we routinely use FACS for maintenance of established cell lines to remove differentiated cells and to dispense graded numbers of highly purified CD13^NEG^SSEA4^POS^Tra-1-60^POS^ populations cells for use in high-throughput derivation and screening assays which include directed differentiation and automated drug screening and phenotyping experiments. This is an important property because the results of these assays could be unequivocally attributed to a defined population of reprogrammed cells rather than to a heterogeneous mixture of cells. Taken together, these results suggest that isolation of the CD13^NEG^SSEA4^POS^Tra-1-60^POS^ population following reprogramming, including integrating or non-integrating viral technologies, allows for the rapid isolation of high quality iPSC lines. Negative selection against CD13^POS^ cells significantly reduces the appearance of transformed cells in ipsc cultures and suggests that negative selection for a marker present on the starting somatic cells can be used to exclude non-reprogrammed or transformed cells from the cultures. Future studies will be needed to determine if this strategy applies to derivation from other somatic cell types or reprogramming methods.

## Supporting Information

Figure S1 **Time Course analysis of retroviral reprogrammed fibroblasts.** The 0825 foreskin fibroblast line was analyzed for changes in pluripotent surface marker expression by flow cytometry at ∼7 dpi intervals following retroviral reprogramming to determine earliest time point at which the CD13^NEG^SSEA4^POS^Tra-1-60^POS^ population appears. Values indicate percent of total cells in the culture expressing the indicated markers.
(TIF)

Figure S2 **Karyotype of FACS and Manually Derived retroviral iPS lines possess a normal karyotype and match the parent fibroblast.** Karyotype was assessed using 20 G-banded metaphase cells from each fibroblast and reprogrammed lines at passages indicated. All lines possess a normal karyotype and match the parent fibroblast. Karyotype was assessed using 20 G-banded metaphase cells from each fibroblast and reprogrammed lines at passages indicated. Fibroblasts and FACS derived lines possess a normal karyotype and match the parent fibroblast. Three out of 20 cells from the manually derived line displayed an unbalanced translocation between the short arm of chromosomes 11 and 22 resulting in trisomy of the short arm of chromosome 11.
(TIF)

Figure S3 **FACS and Manually Derived Sendai iPS lines express pluripotency markers.** FACS **(A)** or Manually **(B)** derived clones were expanded on MEF feeder layers and stained for two common markers of pluripotency: Tra-1-60 and Nanog. 10× Magnification**.** All lines show consistent expression of pluripotency markers. **(C)** qRTPCR showing expression of endogenous gene expression and silencing **(D)** of retroviral genes.
(TIF)

Table S1 **Quantitative real-time PCR Primers.**
(DOC)

Table S2 **Southern Blot Primers.**
(DOC)

Table S3 **NanoString Pluripotency Codeset.**
(DOC)

Table S4 **NanoString Lineage Codeset.**
(DOC)

Table S5 **Primary Antibodies for Immunofluorescence.**
(DOC)

Table S6 **Summary of FACS Derived hIPSC Lines.**
(DOC)

Table S7 **Full Nanostring Data Set For Pluripotent Gene Expresion of Retrovirally Reproggrammed Fibroblasts.**
(XLSX)

Table S8 **Full Nanostring Data Set For Embryoid Bodies Derived From Retrovirally Reproggrammed Fibroblasts.**
(XLSX)

Table S9 **Full Nanostring Data Set For Pluripotent Gene Expresion of Sendai Reproggrammed Fibroblasts.**
(XLSX)

Table S10 **Full Nanostring Data Set For Embryoid Bodies Derived From Sendai Reproggrammed Fibroblasts.**
(XLSX)
