# Supplementary figures and images for: Improved Methods for Reprogramming Human Dermal Fibroblasts Using Fluorescence Activated Cell Sorting
Source: PLoS One. 2013 Mar 29;8(3):e59867. doi: 10.1371/journal.pone.0059867 (PMC3612089; doi:10.1371/journal.pone.0059867)

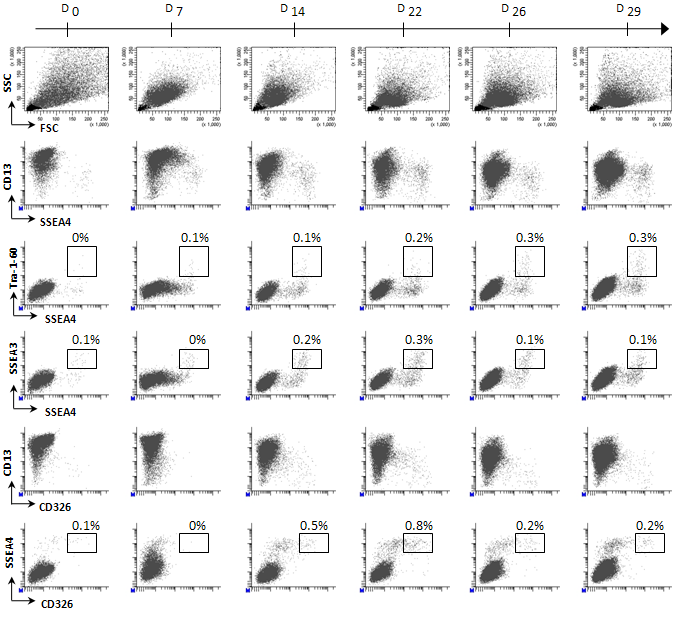

Supplement: Figure S1 — Time Course analysis of retroviral reprogrammed fibroblasts. The 0825 foreskin fibroblast line was analyzed for changes in pluripotent surface marker expression by flow cytometry at ∼7 dpi intervals following retroviral reprogramming to determine earliest time point at which the CD13NEGSSEA4POSTra-1-60POS population appears. Values indicate percent of total cells in the culture expressing the indicated markers. (TIF) [file pone.0059867.s001.tif]

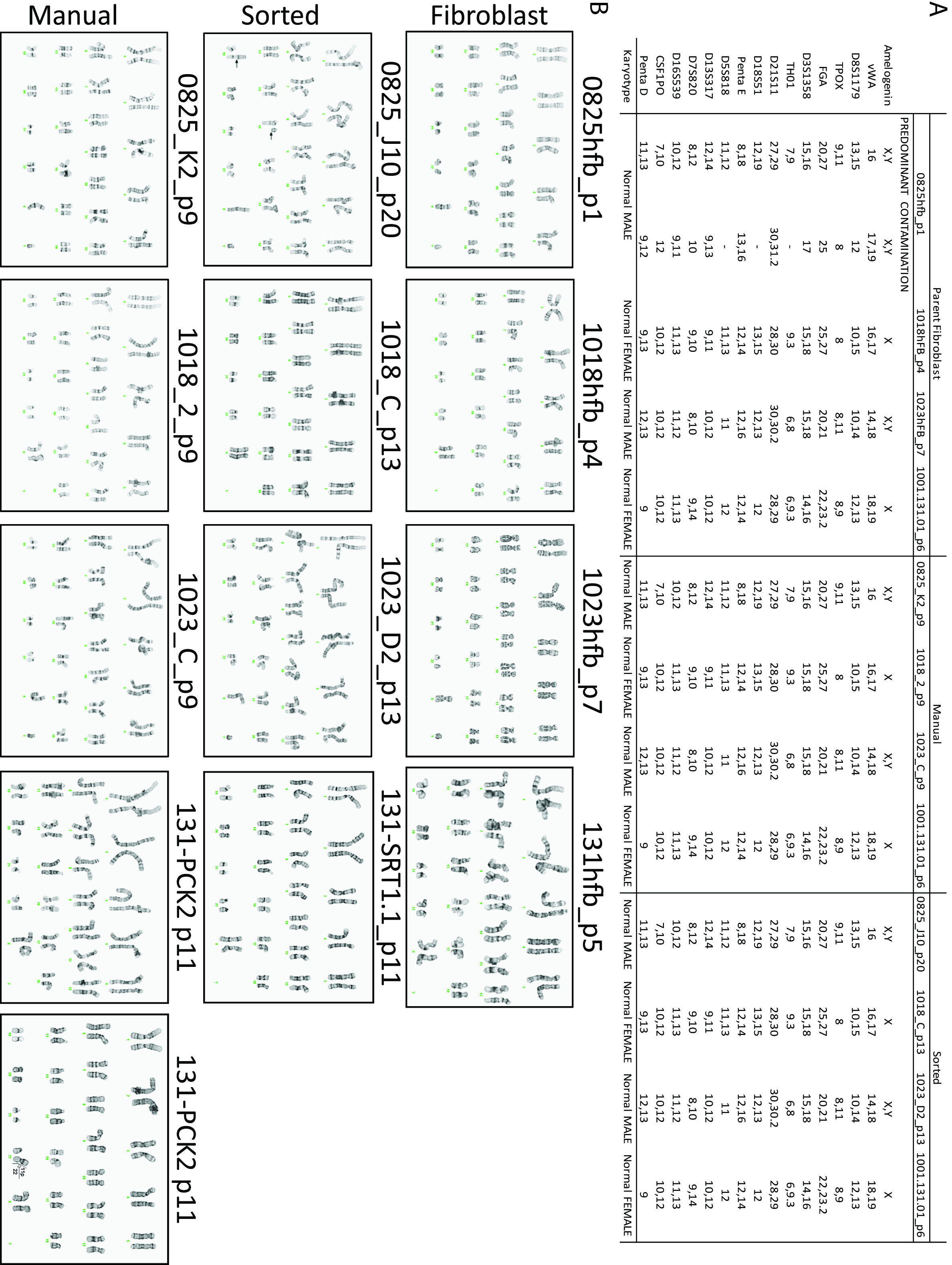

Supplement: Figure S2 — Karyotype of FACS and Manually Derived retroviral iPS lines possess a normal karyotype and match the parent fibroblast. Karyotype was assessed using 20 G-banded metaphase cells from each fibroblast and reprogrammed lines at passages indicated. All lines possess a normal karyotype and match the parent fibroblast. Karyotype was assessed using 20 G-banded metaphase cells from each fibroblast and reprogrammed lines at passages indicated. Fibroblasts and FACS derived lines possess a normal karyotype and match the parent fibroblast. Three out of 20 cells from the manually derived line displayed an unbalanced translocation between the short arm of chromosomes 11 and 22 resulting in trisomy of the short arm of chromosome 11. (TIF) [file pone.0059867.s002.tif]

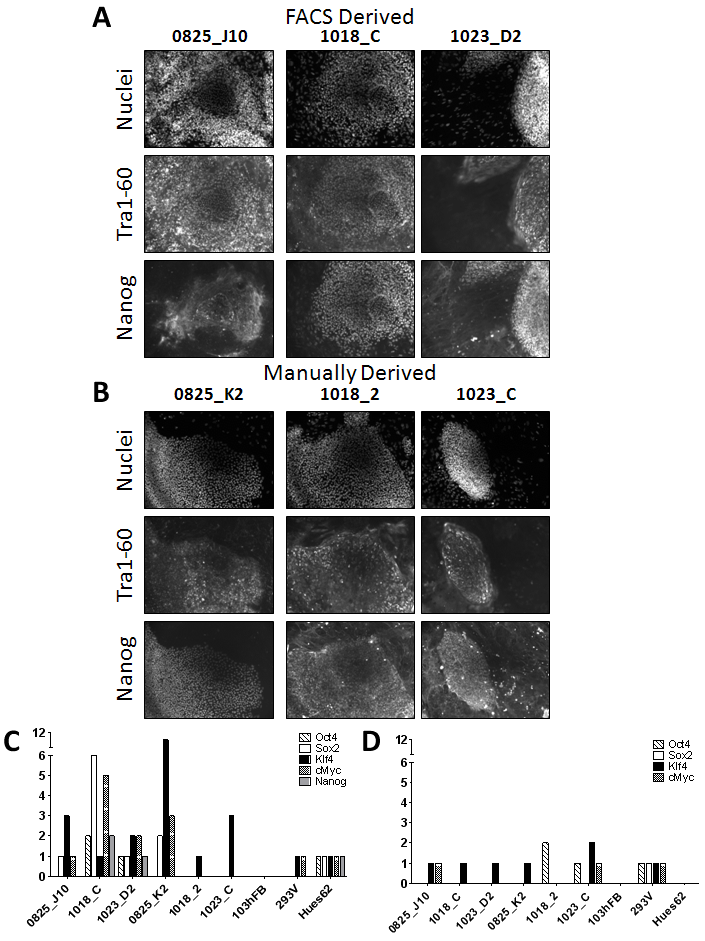

Supplement: Figure S3 — FACS and Manually Derived Sendai iPS lines express pluripotency markers. FACS (A) or Manually (B) derived clones were expanded on MEF feeder layers and stained for two common markers of pluripotency: Tra-1-60 and Nanog. 10× Magnification. All lines show consistent expression of pluripotency markers. (C) qRTPCR showing expression of endogenous gene expression and silencing (D) of retroviral genes. (TIF) [file pone.0059867.s003.tif]
