# Supplementary material for: Improved Methods for Reprogramming Human Dermal Fibroblasts Using Fluorescence Activated Cell Sorting
Source: PLoS One. 2013 Mar 29;8(3):e59867. doi: 10.1371/journal.pone.0059867 (PMC3612089; doi:10.1371/journal.pone.0059867)
Supplement: Table S1 — Quantitative real-time PCR Primers. (DOC) [file pone.0059867.s004.doc]

**Table S1: Quantitative real-time PCR Primers**

| **GENE** | **FORWARD PRIMER 5’-3’** | **REVERSE PRIMER 5’-3’** |
| --- | --- | --- |
| Oct 4 (endogenous) | CCCCAGGGCCCCATTTTGGTACC | GGCACAAACTCCAGGTTTTC |
| Sox2 (endogenous) | ACACTGCCCCTCTCACACAT | GGGTTTTCTCCATGCTGTTTCT |
| Klf4 (endogenous) | ACCCACACAGGTGAGAAACCTT | GTTGGGAACTTGACCATGATTG |
| C-Myc (endogenous) | AGCAGAGGAGCAAAAGCTCATT | CCAAAGTCCAATTTGAGGCAGT |
| Oct4 (transgene) | CCCCAGGGCCCCATTTTGGTACC | AACCTACAGGTGGGGTCTTTCA |
| Sox2 (transgene) | ACACTGCCCCTCTCACACAT | AACCTACAGGTGGGGTCTTTCA |
| Klf4 (transgene) | GACCACCTCGCCTTACACAT | AACCTACAGGTGGGGTCTTTCA |
| C-Myc (transgene) | AGCAGAGGAGCAAAAGCTCATT | AACCTACAGGTGGGGTCTTTCA |
| B2M | TAGCTGTGCTCGGGCTACT | TCTCTGCTGGATGACGCG |
