# Supplementary material for: Improved Methods for Reprogramming Human Dermal Fibroblasts Using Fluorescence Activated Cell Sorting
Source: PLoS One. 2013 Mar 29;8(3):e59867. doi: 10.1371/journal.pone.0059867 (PMC3612089; doi:10.1371/journal.pone.0059867)
Supplement: Table S2 — Southern Blot Primers. (DOC) [file pone.0059867.s005.doc]

| **GENE** | **FORWARD PRIMER 5’-3’** | **REVERSE PRIMER 5’-3’** |
| --- | --- | --- |
| Oct 4 (endogenous) | GAGAAGGAGAAGCTGGAGCA | GTGAAGTGAGGGCTCCCATA |
| Sox2 (endogenous) | AGAACCCCAAGATGCACAAC | TGGAGTGGGAGGAAGAGGTA |
| Klf4 (endogenous) | ACCTGGCGAGTCTGACATGG | TCTTCATGTGTAAGGCGAGGTGG |

**Table S2: Southern Blot Primers**
