# Supplementary material for: Improved Methods for Reprogramming Human Dermal Fibroblasts Using Fluorescence Activated Cell Sorting
Source: PLoS One. 2013 Mar 29;8(3):e59867. doi: 10.1371/journal.pone.0059867 (PMC3612089; doi:10.1371/journal.pone.0059867)
Supplement: Table S3 — NanoString Pluripotency Codeset. (DOC) [file pone.0059867.s006.doc]

**Table S3: NanoString Pluripotency Codeset**

| **Retroviral** | **Sendai transgenes** | **Pluripotency Markers** | **Spontaneous Differentiation** | **Fibroblasts** | **Housekeeping** |
| --- | --- | --- | --- | --- | --- |
| tOct4 | S-tOct4 | POU5F1 (OCT4) | SOX17 | ANPEP (CD13) | ACTB |
| tSox2 | S-tKlf4 | SOX2 | AFP |  | POLR2A |
| tKlf4 | S-tC-myc | KLF4 | NR2F2 |  | ALAS1 |
| tC-Myc | S-tSox2 | MYC |  |  |  |
|  | SeV | LIN28 |  |  |  |
|  |  | NANOG |  |  |  |
|  |  | ZFP42 |  |  |  |
