# Supplementary material for: Improved Methods for Reprogramming Human Dermal Fibroblasts Using Fluorescence Activated Cell Sorting
Source: PLoS One. 2013 Mar 29;8(3):e59867. doi: 10.1371/journal.pone.0059867 (PMC3612089; doi:10.1371/journal.pone.0059867)
Supplement: Table S4 — NanoString Lineage Codeset. (DOC) [file pone.0059867.s007.doc]

**Table S4:** NanoString Lineage Codeset

| **Mesoderm** | **Ectoderm** | **Endoderm** | **Retroviral** | **Sendai** | **Pluripotent** | **Other** | **Housekeeping** |
| --- | --- | --- | --- | --- | --- | --- | --- |
| ABCG2 | ABCG2 | APOE | tOct4 | tOct4 | POU5F1 | SRY | ACTB |
| ADIPOQ | APOE | CD44 | tSox2 | tSox2 | NANOG | XIST | POLR2A |
| ANPEP | CD44 | CDH2 | tKlf4 | tKlf4 | ZFP42 |  | ALAS1 |
| CD34 | CDH2 | CDX2 | tC-Myc | tC-Myc |  |  |  |
| CD36 | CRABP2 | CTNNB1 |  | SeV |  |  |  |
| CD4 | EN1 | FOXA2 |  |  |  |  |  |
| CD44 | FAS | GATA4 |  |  |  |  |  |
| CDH1 | FGFR2 | GATA6 |  |  |  |  |  |
| CDH2 | FUT4 | GCG |  |  |  |  |  |
| CDH5 | GATA2 | HNF1A |  |  |  |  |  |
| CEACAM1 | GATA3 | HNF1B |  |  |  |  |  |
| DLL1 | HAND1 | ISL1 |  |  |  |  |  |
| FUT4 | ICAM1 | ITGA6 |  |  |  |  |  |
| GATA3 | ITGA4 | ITGB1 |  |  |  |  |  |
| GATA4 | ITGA6 | NEUROG3 |  |  |  |  |  |
| HHEX | ITGB1 | NKX2-5 |  |  |  |  |  |
| ICAM1 | MAP2 | PAX6 |  |  |  |  |  |
| INHBA | MAPT | PDX1 |  |  |  |  |  |
| ITGA4 | MCAM | SLC2A2 |  |  |  |  |  |
| ITGA6 | MNX1 | SST |  |  |  |  |  |
| ITGAL | NCAM1 | SYP |  |  |  |  |  |
| ITGAM | NEFL | THY1 |  |  |  |  |  |
| ITGAV | NES |  |  |  |  |  |  |
| ITGAX | NEUROG3 |  |  |  |  |  |  |
| ITGB1 | NGFR |  |  |  |  |  |  |
| ITGB3 | NOG |  |  |  |  |  |  |
| KDR | NOTCH1 |  |  |  |  |  |  |
| KIT | OTX2 |  |  |  |  |  |  |
| LEF1 | PAX3 |  |  |  |  |  |  |
| MCAM | PAX6 |  |  |  |  |  |  |
| MME | PAX7 |  |  |  |  |  |  |
| MYOD1 | PDGFRA |  |  |  |  |  |  |
| MYOG | SNAI2 |  |  |  |  |  |  |
| NCAM1 | SOX10 |  |  |  |  |  |  |
| NES | SOX2 |  |  |  |  |  |  |
| NGFR | SOX9 |  |  |  |  |  |  |
| NOTCH1 | SYP |  |  |  |  |  |  |
| PECAM1 | TDGF1 |  |  |  |  |  |  |
| SDC1 | TH |  |  |  |  |  |  |
| SPI1 | THY1 |  |  |  |  |  |  |
| SRF |  |  |  |  |  |  |  |
| STAT3 |  |  |  |  |  |  |  |
| T |  |  |  |  |  |  |  |
| THY1 |  |  |  |  |  |  |  |
| TNFRSF1A |  |  |  |  |  |  |  |
| TWIST1 |  |  |  |  |  |  |  |
