# Supplementary material for: Improved Methods for Reprogramming Human Dermal Fibroblasts Using Fluorescence Activated Cell Sorting
Source: PLoS One. 2013 Mar 29;8(3):e59867. doi: 10.1371/journal.pone.0059867 (PMC3612089; doi:10.1371/journal.pone.0059867)
Supplement: Table S5 — Primary Antibodies for Immunofluorescence. (DOC) [file pone.0059867.s008.doc]

**Table S5: Primary Antibodies for Immunofluorescence**

| **Antibody** | **Company** | **Catalog #** | **Dilution** |
| --- | --- | --- | --- |
| Oct4 | Stemgent | 09-0023 | 1:250 |
| Sox2 | Stemgent | 09-0024 | 1:250 |
| Tra-1-60 | Millipore | MAB4381 | 1:250 |
| SSEA4 | R&D Systems | MAB1435 | 1:250 |
| Nanog | R&D Systems | AF1997 | 1:250 |
| SSEA3 | R&D Systems | MAB1434 | 1:250 |
| Smooth Muscle | DAKO | M085101 | 1:500 |
| Alpha-1-Fetoprotein | DAKO | A0502 | 1:500 |
| TUJ1 | Covance | MMS-435P | 1:500 |
| Nestin | Millipore | AB5922 | 1:500 |
| MAP2 | Abcam | ab32454 | 1:500 |
