# Supplementary material for: Improved Methods for Reprogramming Human Dermal Fibroblasts Using Fluorescence Activated Cell Sorting
Source: PLoS One. 2013 Mar 29;8(3):e59867. doi: 10.1371/journal.pone.0059867 (PMC3612089; doi:10.1371/journal.pone.0059867)
Supplement: Table S6 — Summary of FACS Derived hIPSC Lines. (DOC) [file pone.0059867.s009.doc]

**Table S6: Summary of FACS Derived hIPSC Lines**

| **Model*** | **Cell Lines** | **Total Derivations** | **Retro** | **Sendai** |
| --- | --- | --- | --- | --- |
| Alzheimer | 11 | 20 | 19 | 1 |
| Parkinsons | 4 | 8 | 2 | 6 |
| FTD | 2 | 2 | 0 | 2 |
| GAN | 5 | 14 | 14 | 0 |
| Cardiac_LMNA | 3 | 7 | 6 | 1 |
| Cardiac_LongQT | 6 | 14 | 14 | 0 |
| MODY | 11 | 34 | 24 | 10 |
| T1D | 3 | 24 | 17 | 7 |
| T2D | 1 | 8 | 8 | 0 |
| MS_RR | 1 | 2 | 0 | 2 |
| MS_SP | 1 | 1 | 0 | 1 |
| Control | 28 | 94 | 51 | 43 |
| Totals | 76 | 228 | 155 | 73 |

*****MS_RR Multiple Sclerosis Relapsing Remitting, MS_SP Multiple Sclerosis Secondary Progressive, FTD Frontal Temporal Dementia, GAN Giant Axonal Neuropathy, LMNA Lamin A/C, MODY Mature Onset Diabetes of the Young
